# Dosimetric investigation of whole-brain radiotherapy with helical intensity modulated radiation therapy and volumetric modulated arc therapy for scalp sparing

**DOI:** 10.1259/bjro.20220037

**Published:** 2023-03-22

**Authors:** Ryosuke Shirata, Tatsuya Inoue, Satoru Sugimoto, Anneyuko I Saito, Motoko Omura, Yumiko Minagawa, Keisuke Sasai

**Affiliations:** 1 Department of Radiation Oncology, Graduate School of Medicine, Juntendo University, 2-1-1 Hongo, Bunkyo-ku, Tokyo, Japan; 2 Department of Radiation Oncology, Shonan Kamakura General Hospital, 1370-1 Okamoto, Kamakura, Kanagawa, Japan; 3 Department of Radiation Oncology, Juntendo University, 2-1-1 Hongo, Bunkyo-ku, Tokyo, Japan

## Abstract

**Objective:**

Intensity-modulated radiotherapy (IMRT) is a well-established radiotherapy technique for delivering radiation to cancer with high conformity while sparing the surrounding normal tissue. Two main purposes of this study are: (1) to investigate dose calculation accuracy of helical IMRT (HIMRT) and volumetric-modulated arc therapy (VMAT) on surface region and (2) to evaluate the dosimetric efficacy of HIMRT and VMAT for scalp-sparing in whole brain radiotherapy (WBRT).

**Methods:**

First, using a radiochromic film and water-equivalent phantom with three types of boluses (1, 3, 5 mm), calculation/measurement dose agreement at the surface region in the VMAT and HIMRT plans were examined. Then, HIMRT, 6MV-VMAT and 10MV-VMAT with scalp-sparing, and two conventional three-dimensional conformal radiotherapy plans (6MV-3DCRT and 10MV-3DCRT; as reference data) were created for 30 patients with brain metastasis (30 Gy/10 fractions). The mean dose to the scalp and the scalp volume receiving 24 and 30 Gy were compared.

**Results:**

The percentage dose differences between the calculation and measurement were within 7%, except for the HIMRT plan at a depth of 1 mm. The averaged mean scalp doses [Gy], V24Gy [%], and V30Gy [%] (1SD) for 6MV-3DCRT, 10MV-3DCRT, HIMRT, 6MV-VMAT, and 10MV-VMAT were [26.6 (1.1), 86.4 (7.3), 13.2 (4.2)], [25.4 (1.0), 77.8 (7.5), 13.2 (4.2)], [23.2 (1.5), 42.8 (19.2), 0.2 (0.5)], [23.6 (1.6), 47.5 (17.9), 1.2 (1.8)], and [22.7 (1.7), 36.4 (17.6), 0.7 (1.1)], respectively.

**Conclusion:**

Regarding the dose parameters, HIMRT achieved a lower scalp dose compared with 6MV-VMAT. However, the highest ability to reduce the mean scalp dose was showed in 10MV-VMAT.

**Advances in knowledge:**

Scalp-sparing WBRT using HIMRT or VMAT may prevent radiation-induced alopecia in patients with BM.

## Introduction

Brain metastasis (BM) accounts for 30% of all intracranial tumors, and approximately 20% of all patients with cancer are estimated to develop BM during their disease.^1^ Current treatment options for BM include surgery, chemotherapy, supportive care, and radiotherapy. An option is fundamentally selected based on the patient’s prognostic factors, such as age, Karnofsky performance score, and number and size of the BM.^2^ Whole brain radiotherapy (WBRT), standard palliative treatment for BM, potentially extends the median survival time from 1 to 4 months or longer by controlling the intracranial micrometastases and providing the palliation of neurological debilitating symptoms.^3,4^ Side effects after WBRT include alopecia, neurocognitive decline, xerostomia, and otitis.^5,6^ These symptoms frequently exert a negative impact on the patient’s health-related quality of life (QOL).

Advanced radiation delivery techniques, such as intensity-modulated radiationtherapy (IMRT), volumetric-modulated arc therapy (VMAT), and helical tomotherapy (HIMRT) have been reported to be able to achieve better dose conformity to the target volume and reduce the dose to the organs at risk (OARs) for many treatment sites than conventional three-dimensional conformal radiotherapy (3DCRT). Hence, they have been applied to WBRT to prevent the side-effects. For instance, a previous planning study has reported that HIMRT plans in WBRT achieved conformal hippocampus-sparing with homogeneous whole brain dose distribution.^7^ Furthermore, clinical trials were conducted with the IMRT technique to spare hippocampal irradiation and decrease neurocognitive deterioration (RTOG 0933, NRG-CC001).^8,9^ The trials showed that reducing the dose to the hippocampus while sustaining uniform dose delivery to the remaining part of the brain can prevent a serious decline in the cognitive function. Radiation-induced acute and chronic alopecia are well-known undesirable side-effects of WBRT.^10^ Apart from the hippocampus, several studies to alleviate the scalp dose for preventing alopecia have been conducted with the IMRT techniques.^5,11–16^ If the mean scalp dose was limited to a range of 16–18 Gy (the prescription dose is 30 Gy in 10 fractions), it resulted in a short period of temporary alopecia and possibly reduced the risk of permanent alopecia.^16^ Alternatively, another retrospective cohort study has assessed the characteristics of persistent alopecia in primary central nervous system tumors or head and neck sarcoma patients after cranial radiotherapy. It showed that 36.1 Gy was the maximum scalp dose, at which Grade 2 alopecia was 50% likely going to occur. The dose is comparable to 30 Gy in 10 fractions if α/β ratiois 3 Gy for late toxicity.^17^

However, a consensus on the relationship between alopecia and scalp dose in WBRT has not been established because of limited data available. Therefore, we are conducting a multi-institutional cohort study to provide informative data on alopecia after WBRT. Accordingly, further investigation of the feasibility of scalp-sparing in advanced radiation delivery techniques is necessary because the treatment planning process is challenging. IMRT and VMAT have dosimetric advantages over 3DCRT in terms of scalp-sparing, furthermore a few studies indicated the possibility that IMRT with a helical technique or high beam energy has a considerable impact on scalp-sparing.^18,19^ This study demonstrated the dosimetric characteristics of the two types of IMRT techniques, VMAT and HIMRT in WBRT for scalp-sparing in a planning study. Furthermore, a phantom study with film dosimetry was performed to evaluate the dose calculation accuracy for the surface region in the radiation treatment planning system (RTPS).

## Methods and materials

### Phantom study

#### Phantom data and structure definition

**Figure 1.**
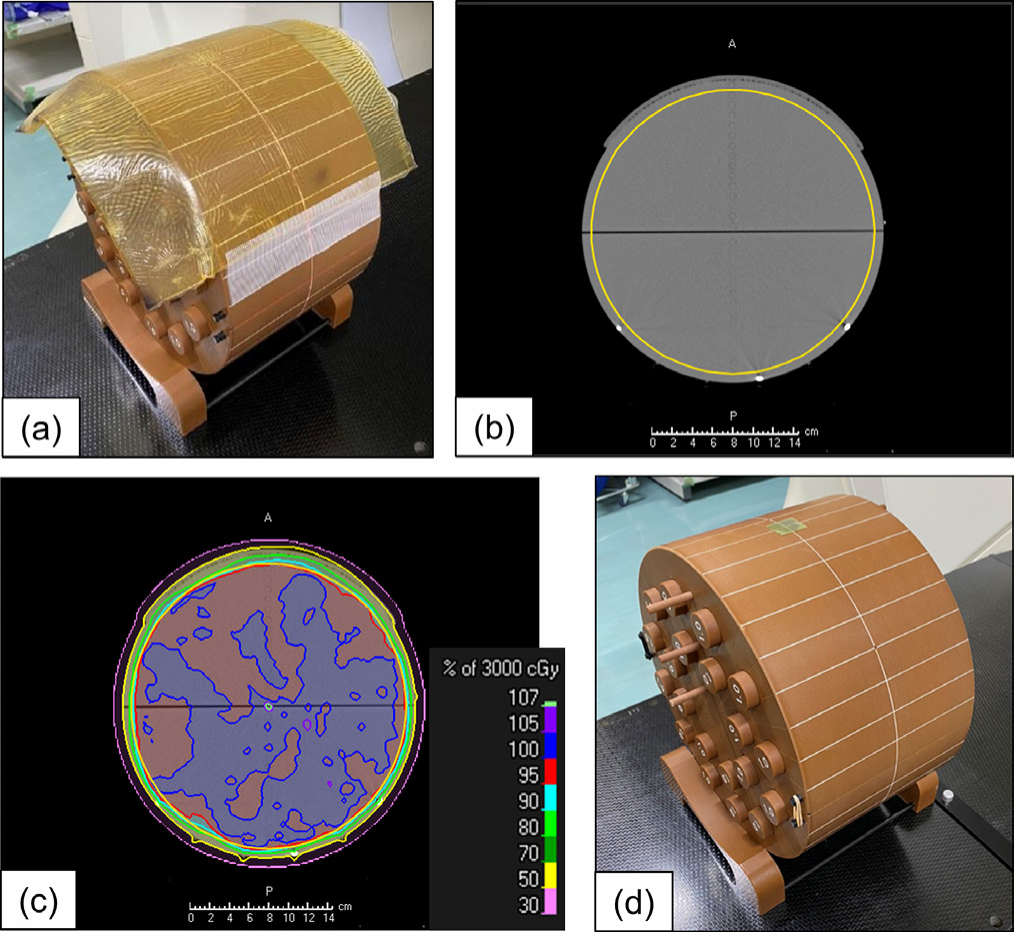
Setup for the surface dose measurements. (**a**) Water-equivalent solid phantom with a 5 mm bolus, (**b**) CT image with the target ROI (yellow line), (**c**) planned dose distribution using 10MV-VMAT, and (**d**) water-equivalent solid phantom with a piece of the Gafchromic EBT3 film. ROI, region of interest; VMAT, volumetric-modulated arc therapy.

A phantom study with a cylindrical water-equivalent solid phantom with a radius of 15.0 cm (Cheese phantom; GammexRMI, Middleton, WI) and Gafchromic EBT3 films (International Specialty Product, NJ) was performed to evaluate the conformity between the calculated and actual doses at the surface region in the VMAT and HIMRT plans. Three CTs having images of the phantom with a bolus (thicknesses of 1, 3, and 5 mm) were acquired using a multidetector-row CT scanner (Aquilion LB, Canon Medical Systems, Tochigi, Japan) with a slice thickness of 2 mm. Figure 1a shows the CT acquisition setup of the phantom with a 5 mm bolus. Subsequently, the CT images were imported to a MIM software system (v. 6.7.1; MIMvista Corp., Cleveland, OH), and a target region of interest (ROI) was created on the CT images. This ROI was cylindrical with a diameter and length of 28 and 5 cm, respectively (Figure 1b).

### Treatment planning

The HIMRT with a beam energy of 6 MV and VMAT with 6- or 10 MV treatment plans were created for each CT image. The plans were optimized to achieve a 95% target coverage with at least 95% of the prescription dose of 30 Gy in 10 fractions. The target dose was maintained between the minimum dose 27 Gy and the maximum dose 31 Gy. The dose to the bolus was set to the maximum dose value of 0.01 Gy. Optimization parameters were empirically selected such that the dose to the bolus achieved the minimal while maintaining the target coverage. Table 1 contains the optimization parameters used for the HIMRT and VMAT plans. The HIMRT plans were created using Planning Station RTPS (v. 5.1.2, Accuray, Madison, WI) commissioned for TomoTherapy Hi-Art (Accuray, Madison, WI). The calculation parameters were a 1.05 cm field width, 0.43 pitch, and 2.0 modulation factor.^20^ The final dose after 250 iterations was calculated using the Collapsed Cone Convolution (CCC) algorithm with 2mm-dose grid size. Using Eclipse RTPS (v. 13.6, Varian Medical Systems, Palo Alto, CA) commissioned for Varian TrueBeam equipped with millennium 120 multileaf collimator (MLC), the VMAT plans were optimized with two full arcs. The collimator angles for counterclockwise and clockwise arcs were 10° and 350°, respectively, to reduce the tongue and groove effect and the effect of interleaf leakage.^21^ The anisotropic analytical algorithm (AAA) was used for the dose calculation with heterogeneous correction and a grid resolution of 2 mm. Figure 1c shows the dose distribution in the VMAT-10MV plan for the phantom with a 5 mm bolus.

**Table 1.** Optimization parameters for VMAT and HIMRT treatmnet planning

Phantom study	VMAT				HIMRT							
	Type	DVH volume (%)	DVH dose (Gy)	Priority	Importance	Max dose (Gy)	Max dose penalty	DVH volume (%)	DVH dose (Gy)	DVH penalty	Min dose (Gy)	Min dose penalty
Target	Lower	100	27.0	600	600	31.0	13	95	28.5	10	27.0	10
	Lower	95	28.5	600	600	31.0	13	50	30.0	10	27.0	10
	Lower	50	30.0	600	600	31.0	13	2	30.5	10	27.0	10
	Upper	2	30.5	600								
	Upper	0	31.0	600								
Bolus	Upper	1	0.01	4	4	30.0	10	0.01	0.01	10		
												
Planning study												
CTV	Lower	100	27.0	600	600	31.0	13	95	28.5	10	27.0	10
	Lower	95	28.5	600	600	31.0	13	50	30.0	10	27.0	10
	Lower	50	30.0	600	600	31.0	13	2	30.5	10	27.0	10
	Upper	2	30.5	600								
	Upper	0	31.0	600								
PTV	Lower	100	27.0	600	600	31.0	13	95	28.5	10	27.0	10
	Lower	95	28.5	600	600	31.0	13	50	30.0	10	27.0	10
	Lower	50	30.0	600	600	31.0	13	2	30.5	10	27.0	10
	Upper	2	30.5	600								
	Upper	0	31.0	600								
R-Lens	Upper	2	8.0	2	2	8.0	8	1	8.0	8		
	Upper	0	8.0	2								
L-Lens	Upper	2	8.0	2	2	8.0	8	1	8.0	8		
	Upper	0	8.0	2								
Scalp (3 mm)	Upper	1	0.01	4	4	30.0	10	0.01	0.01	10		
Scalp (5 mm)	Upper	1	0.01	4	4	30.0	10	0.01	0.01	10		

CTV, clinical target volume; DVH, dose–volume histogram; HIMRT, helical intensity-modulated radiotherapy; VMAT, volumetric-modulated arc therapy.

### Film measurement and analysis

The EBT3 films were cut into small pieces of 2 × 2 cm^2^ and placed atop the phantom (Figure 1d) and covered with the bolus after registering the pre-treatment cone beam CT or mega voltage CT image with the planning kVCT image. Each measurement was performed thrice. In addition, film irradiations were performed using each treatment machine and energy to create the optical density–dose calibration table.^22^ The irradiated films were scanned 24 h after irradiation using an Epson 10000G flatbed scanner (Epson America, Inc., Long Beach, CA) with a resolution of 72 dpi and a 16-bit grayscale format. The film images were saved in TIFF format and analyzed using the DQA Analysis Tool (Accuray, Madison, WI). Each averaged film dose was compared with the RTPS calculated dose averaged over the corresponding region below the bolus (2 × 2 × 0.0278 cm^3^; film size and thickness).

### Planning study

#### Patient data

A data set of 30 consecutive patients (male: 15, female: 15) with BM who underwent WBRT was used for this retrospective planning study. Patient selection criteria was as follows: (1) those who underwent WBRT with a prescription dose of 30 Gy in 10 fractions, (2) adults over 20 years (median age, 70 years [range = 45–86 years]), and (3) those with no history of intracranial surgery. All the patients were previously treated with a linear accelerator (Elekta Synergy Platform) with 40 MLC leaf pairs with a leaf width of 10 mm (Elekta AB, Stockholm, Sweden), using lateral-opposed beams with 6 MV photons at Juntendo University Urayasu Hospital. The treatment plans were created using Pinnacle^3^ (v. 9.10, Philips Healthcare, Andover, MA) commissioned for the linear accelerator. A thermoplastic mask was used to ensure immobilization for the stable positioning of the patients. The CT images were scanned using a two-row CT scanner (GE Healthcare, Madison, WI) and the reconstruction resolution size was 0.967 × 0.967 × 5 mm^3^. This study was approved by our Institutional Review Board (No. TGE01072-024).

### Structure definition

**Figure 2.**
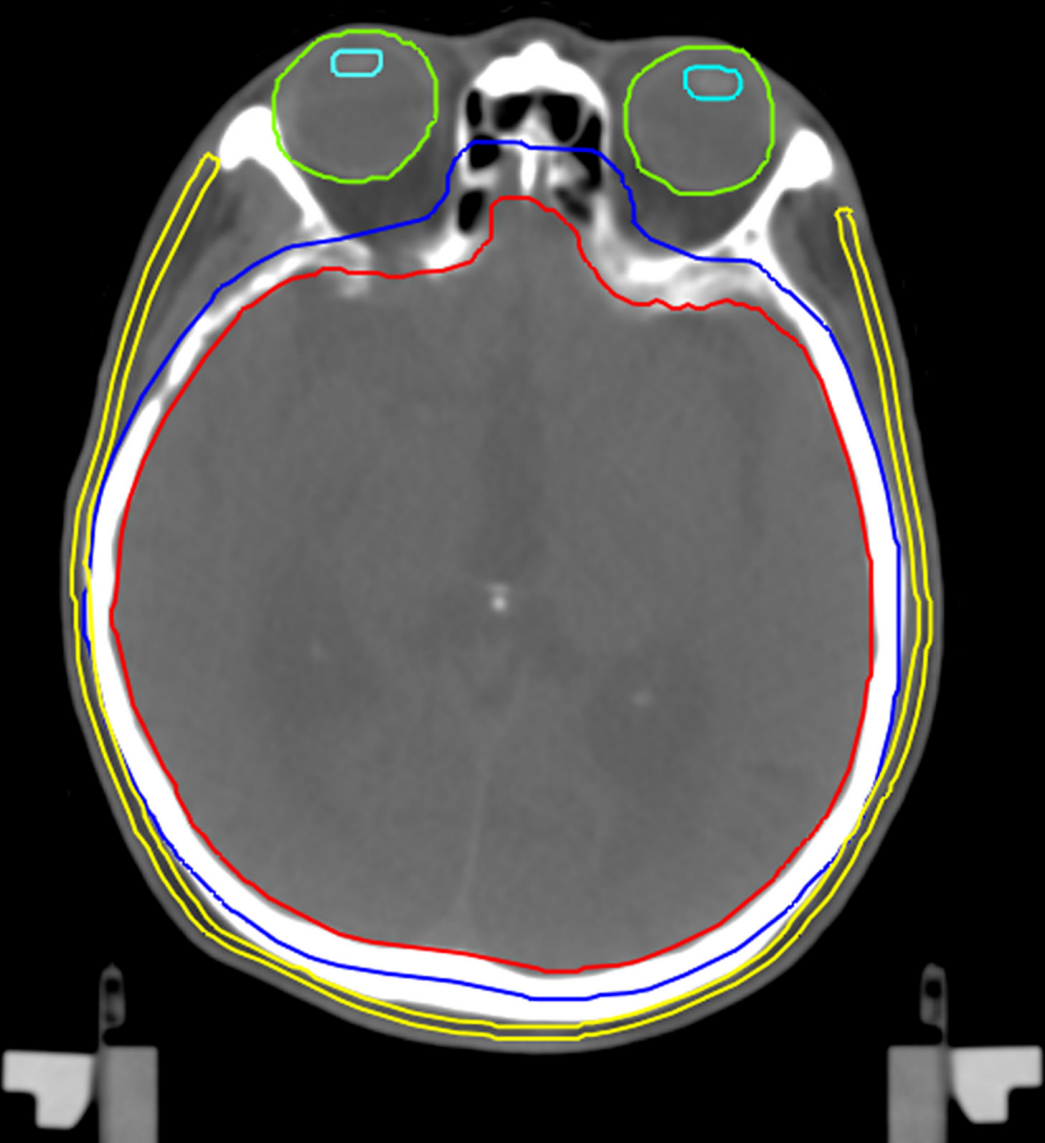
Contoured structures on a CT image. Red and blue lines show the CTV and PTV (5 mm margin), respectively. Yellow, green, and sky-blue lines show the scalp, eyes, and lenses defined as the OARs, respectively. CTV, clinical target volume; OAR, organ at risk; PTV, planning target volume.

**Figure 3.**
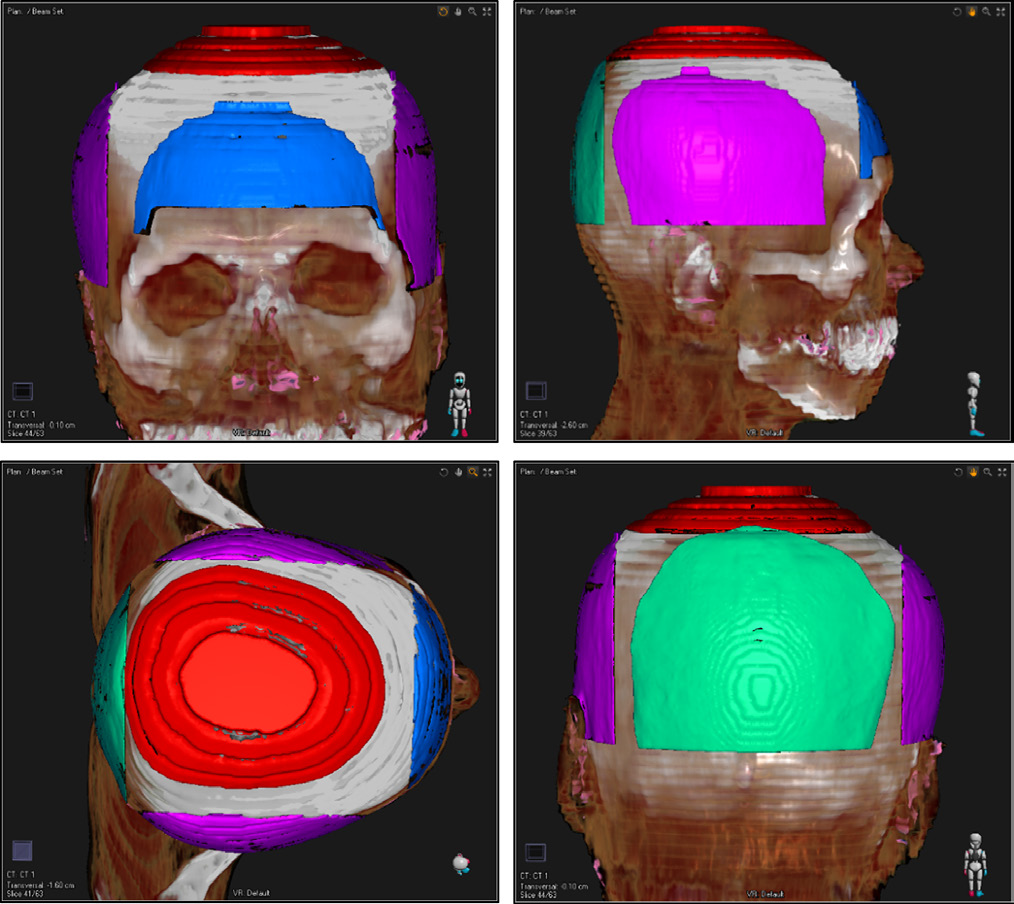
Four subvolumes of the scalp for dosimetric evaluation. Blue, red, green, and purple ROIs show the front, top, back, and lateral regions of the scalp. ROI, region of interest

The whole brain as a clinical target volume (CTV) for the patients was delineated on the planning CT by a radiation oncologist with over 15 years of Pinnacle^3^ experience. The lenses were delineated as OARs. The scalp was defined as the region at a depth from 3 to 5 mm below the skin surface. Figure 2 shows the contoured structures. In addition, six types of planning target volumes (PTVs) were created by isotropically expanding the CTV by a 0–5 mm margin increment in 1 mm thickness to evaluate the impact of the target volume on the surface dose. Furthermore, each scalp structure was divided into four subvolumes, comprising the top, front, lateral, and back regions, to separately evaluate the dose contribution of each irradiation technique. Each subvolume was defined as the volume overlapped between the scalp and a 2 cm perpendicular depth from the cortex in each direction. Figure 3 shows the delineation of the four subvolumes.

### Treatment planning

Five WBRT plans for each patient were created using three different RTPS. These plans comprised 3DCRT with energies of 6 or 10 MV photons, HIMRT with 6 MV, and two coplanar arcs of VMAT with energies of 6 or 10 MV. The plans were created to achieve clinically acceptable PTV coverage (at least 95% of the PTV covered by 95% of the prescription dose)^23^ and lenses sparing (the maximum dose <10 Gy) with the prescription dose of 30 Gy in 10 fractions. Here, 3DCRT plans were created as reference data using two standard lateral-opposed fields with the gantry at 90° and 270° in Pinnacle^3^. The treatment fields were conformed around the PTV with a leaf margin of 5 mm, and the isocenter was positioned at the centroid of the PTV. The lenses were blocked with the MLCs to prevent direct irradiation. The CCC algorithm was used for the dose calculation with a grid resolution of 2 mm. High dose regions (> 107% of the prescription dose) were removed using a field-in-field technique without compromising the PTV coverage. Dose optimization was used for HIMRT and VMAT plans to adhere to the following constraints: dose to 95% of the PTV volume (V95%) >28.5 Gy, maximum dose <31 Gy, and minimum dose >27 Gy for the PTV; maximum dose <8 Gy for the lenses. In addition to PTV, CTV was used in the optimization to achieve homogenous target dose distribution. The scalp dose was decreased as low as feasible by constraining the 3 and 5 mm scalp dose to 0.01 Gy. Table 1 lists the optimization parameters for the target and OARs that were standardly employed for all patients. Similar parameters were chosen with fair planning in mind, although the TPS differs in HIMRT and VMAT. The calculation parameters were the same as those used in the aforementioned phantom study.

### Plan evaluation and statistical analysis

The CT images, structures, and calculated dose distributions were imported to the MIM software system to compare the dosimetric parameters across different RTPSs. Based on the data normality, the paired *t*-test or Wilcoxon signed-rank test was used to statistically compare the differences in the parameters among the 3DCRT, HIMRT, and VMAT plans. A *p*-value <0.01 was considered statistically significant. Statistical analysis was performed using Microsoft Excel (Microsoft, Redmond, WA).

## Results

Table 2 shows the ratios of the average doses of the EBT3 films and RTPS doses at each depth for the HIMRT, 6MV-VMAT, and 10MV-VMAT plans. The ranges of the ratios were 0.98–1.30, 1.02–1.07, and 0.97–1.07 at depths of 1, 3, and 5 mm, respectively. The maximal difference in the measurement and calculated doses was observed at a depth of 1 mm for the HIMRT plan. The percentage differences in the other plans were within 7%.

**Table 2.** Surface doses of the film measurement and TPS calculation and ratios

	1 mm depth			3 mm depth			5 mm depth		
	Film (1SD) [Gy]	TPS [Gy]	Ratio	Film (1SD) [Gy]	TPS [Gy]	Ratio	Film (1SD) [Gy]	TPS [Gy]	Ratio
HIMRT	1.80 (0.01)	1.38	1.30	1.95 (0.02)	1.92	1.02	2.09 (0.06)	2.01	1.04
6MV-VMAT	1.73 (0.01)	1.77	0.98	2.47 (0.02)	2.31	1.07	2.33 (0.02)	2.40	0.97
10MV-VMAT	1.94 (0.01)	1.85	1.05	1.98 (0.03)	1.94	1.02	2.42 (0.05)	2.26	1.07

HIMRT, helical intensity-modulated radiotherapy; VMAT, volumetric-modulated arc therapy.

The clinical goals for PTV and lenses were achieved in all the treatment plans. The average and standard deviations of the PTV D95% were 30.1 ± 0.2 (6MV-3DCRT), 30.2 ± 0.1 (10MV-3DCRT), 28.6 ± 0.1 (HIMRT), 28.7 ± 0.1 (6MV-VMAT), and 28.5 ± 0.1 Gy (10MV-VMAT). The average and standard deviations of the left- and right-lens maximum doses were 6.8 ± 2.2, 6.4 ± 2.7 (6MV-3DCRT); 7.6 ± 2.2, 7.0 ± 2.7 (10MV-3DCRT); 8.4 ± 0.4, 8.3 ± 0.4 (HIMRT); 9.4 ± 1.2, 9.2 ± 1.0 (6MV-VMAT); and 9.6 ± 1.1, 9.5 ± 1.1 Gy (10MV-VMAT).

**Figure 4.**
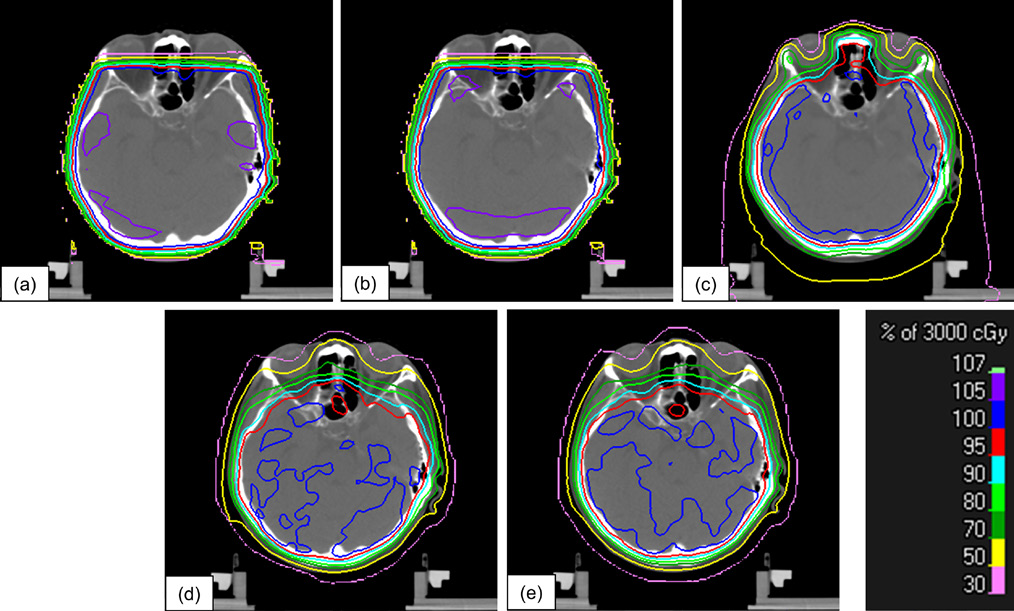
Dose distribution of the five types of treatment plans for a patient. (**a**) 6MV-3DCRT, (**b**) 10MV-3DCRT, (**c**) HIMRT, (**d**) 6MV-VMAT, and (**e**) 10MV-VMAT. The color bar indicates the absorbed dose levels between 30 and 107% of the prescription dose (3000cGy). 3DCRT, three-dimensional conformal radiotherapy; HIMRT, helical intensity-modulated radiotherapy; VMAT, volumetric-modulated arc therapy.

Figure 4 shows the dose distributions of the five treatment plans for a patient. The dose distributions of HIMRT and both VMAT plans exhibited visually high conformity to the target without a high dose region (the percentage volumes of PTV receiving more than 105% of the prescribed dose), compared with those of the 3DCRT plans.

**Figure 5.**
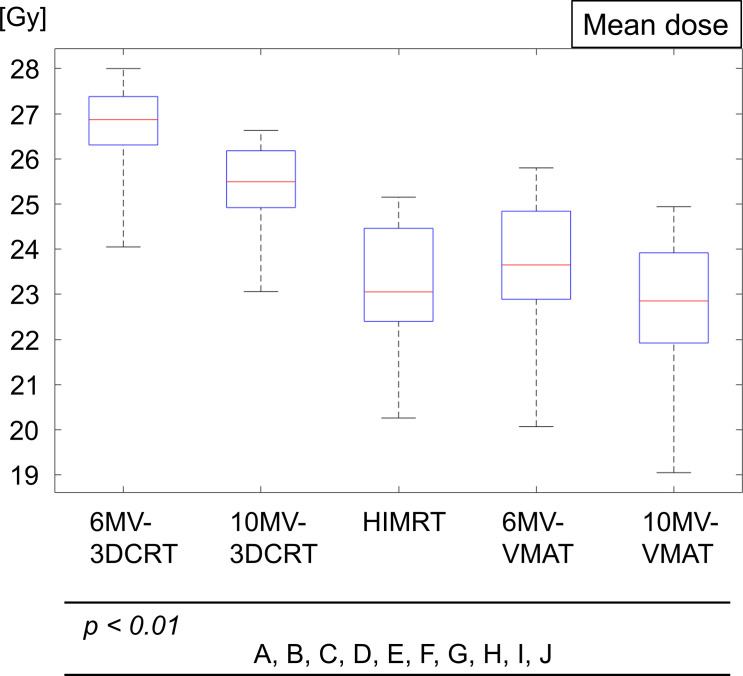
Boxplot showing the mean scalp dose over 30 patients for five treatment plans. Each box comprises the minimum and maximum range values, upper and lower quartiles, and median. The alphabets represent *p* < 0.01, A: 6MV-3DCRT *vs* 10MV-3DCRT; B: 6MV-3DCRT *vs* HIMRT, C: 6MV-3DCRT *vs* 6MV-VMAT; D: 6MV-3DCRT *vs* 10MV-VMAT; E: 10MV-3DCRT *vs* HIMRT; F: 10MV-3DCRT *vs* 6MV-VMAT; G: 10MV-3DCRT *vs* 10MV-VMAT; H: HIMRT *vs* 6MV-VMAT; I: HIMRT *vs* 10MV-VMAT; and J: 6MV-VMAT *vs* 10MV-VMAT.

Figure 5 shows the boxplot of the mean scalp doses for five plans with a 5 mm PTV margin. The average and standard deviations of the mean scalp doses were 26.6 ± 1.1 (6MV-3DCRT), 25.4 ± 1.0 (10MV-3DCRT), 23.2 ± 1.5 (HIMRT), 23.6 ± 1.6 (6MV-VMAT), and 22.7 ± 1.7 Gy (10MV-VMAT). The mean scalp doses of the HIMRT and VMAT plans were smaller than that of the 3DCRT plans (*p* < 0.01). The mean scalp dose of the HIMRT plans was significantly smaller than that of the 6MV-VMAT plans (*p* < 0.01). The mean scalp dose of the 10MV-VMAT plans was the smallest (*p* < 0.01).

**Figure 6.**
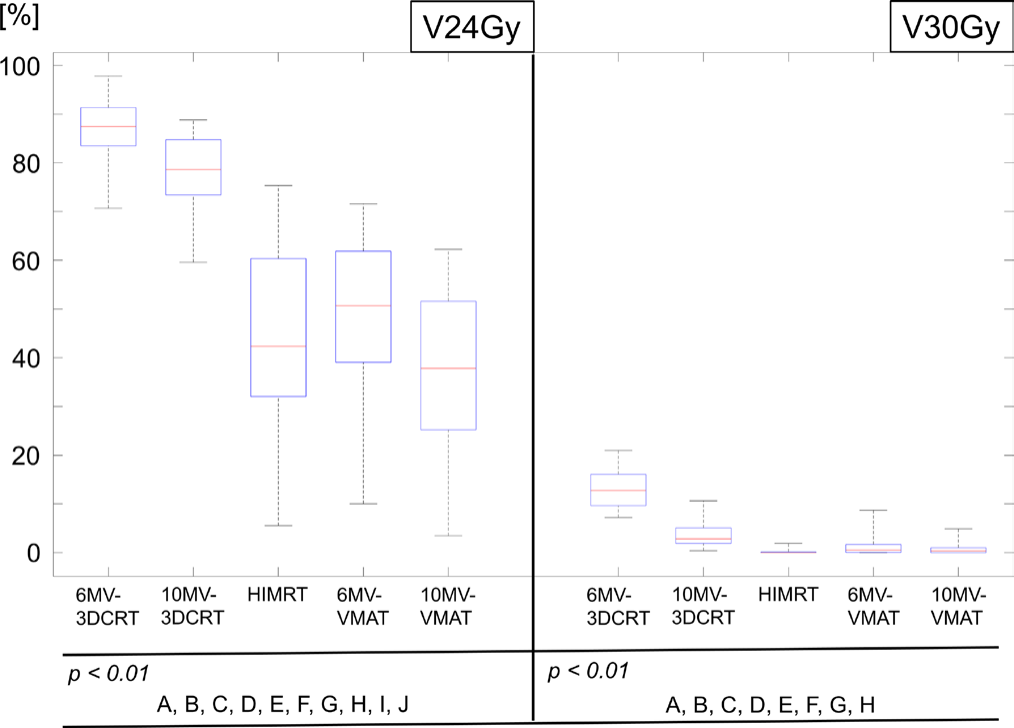
Boxplot showing the V24Gy and V30Gy of the scalp over 30 patients for five treatment plans. Each box comprises the minimum and maximum range values, upper and lower quartiles, and median. The alphabets represent *p* < 0.01, A: 6MV-3DCRT *vs* 10MV-3DCRT; B: 6MV-3DCRT *vs* HIMRT; C: 6MV-3DCRT *vs* 6MV-VMAT; D: 6MV-3DCRT *vs* 10MV-VMAT; E: 10MV-3DCRT *vs* HIMRT; F: 10MV-3DCRT *vs* 6MV-VMAT; G: 10MV-3DCRT *vs* 10MV-VMAT; H: HIMRT *vs* 6MV-VMAT; I: HIMRT *vs* 10MV-VMAT; and J: 6MV-VMAT *vs* 10MV-VMAT. 3DCRT, three-dimensional conformal radiotherapy; HIMRT, helical intensity-modulated radiotherapy; VMAT, volumetric-modulated arc therapy.

The boxplots for the V24Gy and V30Gy of the scalp for the five plans are shown in Figure 6. The average and standard deviations of V24Gy and V30Gy were 86.4 ± 7.3, 13.2 ± 4.2 (6MV-3DCRT); 77.8 ± 7.5, 13.2 ± 4.2 (10MV-3DCRT); 42.8 ± 19.2, 0.2 ± 0.5 (HIMRT); 47.5 ± 17.9, 1.2 ± 1.8 (6MV-VMAT); and 36.4 ± 17.6, 0.7 ± 1.1% (10MV-VMAT), respectively. The statistically significant differences (*p* < 0.01) between any two of the plans were observed for both parameters, except for HIMRT *vs* 10MV-VMAT in V30Gy.

Table 3 shows the average and standard deviations of the mean scalp doses of the five plans when the PTV margin was changed from 0 to 5 mm.

**Table 3.** Average and standard deviations of the mean scalp doses for various PTV margins of the five treatment plans

		Mean dose (1SD)[Gy]				
		6MV-3DCRT	10MV-3DCRT	HIMRT	6MV-VMAT	10MV-VMAT
PTV margin [mm]	5	26.6 (1.1)	25.4 (1.0)	23.2 (1.5)	23.6 (1.6)	22.7 (1.7)
	4			22.4 (1.3)	22.7 (1.4)	21.8 (1.5)
	3			21.7 (1.2)	22.0 (1.4)	21.0 (1.2)
	2			20.0 (1.2)	20.0 (1.2)	19.0 (1.2)
	1			19.7 (1.1)	19.6 (1.1)	18.5 (1.2)
	0	22.5 (1.7)	21.3 (1.4)	18.0 (1.3)	19.1 (1.1)	17.9 (1.1)

3DCRT, three-dimensional conformal radiotherapy; HIMRT, helical intensity-modulated radiotherapy; PTV, planning target volume; VMAT, volumetric-modulated arc therapy.

**Figure 7.**
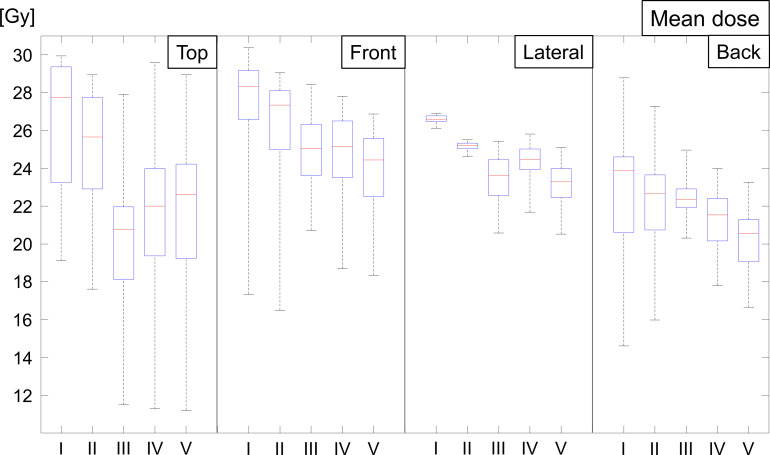
Boxplot showing the mean scalp dose of the four subvolumes over 30 patients for 5 treatment plans. Each box comprises the minimum and maximum range values, upper and lower quartiles, and median. Numbers I, II, III, IV, and V show 6MV-3DCRT, 10MV-3DCRT, HIMRT, 6MV-VMAT, and 10MV-VMAT, respectively. 3DCRT, three-dimensional conformal radiotherapy; HIMRT, helical intensity-modulated radiotherapy; VMAT, volumetric-modulated arc therapy.

Figure 7 shows the boxplot for the mean scalp doses of the four subvolumes. The average and standard deviations of the mean scalp doses of the top, front, lateral, and back regions were 26.3 ± 3.4, 27.2 ± 3.2, 26.6 ± 0.2, 22.8 ± 3.4 (6MV-3DCRT); 24.9 ± 3.3, 26.2 ± 2.9, 25.2 ± 0.2, 21.9 ± 2.9 (10MV-3DCRT); 20.0 ± 4.1, 24.9 ± 1.8, 23.4 ± 1.3, 22.4 ± 1.1 (HIMRT); 21.2 ± 4.5, 24.7 ± 2.1, 24.3 ± 1.1, 21.3 ± 1.6 (6MV-VMAT); and 21.3 ± 4.5, 24.0 ± 2.1, 23.1 ± 1.3, 20.4 ± 1.7 Gy (10MV-VMAT), respectively.

## Discussion

Long-term survival for patients with BM has become possible because of the improvements in treatment schemes. Therefore, it is essential to avoid the side-effects caused by radiotherapy. If the occurrence of radiation-induced alopecia can be prevented, it would greatly improve the QOL of patients, particularly females and children. Here, we demonstrated the efficacy and feasibility of advanced radiation techniques, HIMRT and VMAT, in reducing the scalp dose during WBRT.

At the beginning of this study, film dosimetry was performed to verify the accuracy of the RTPS dose calculation at surface regions. The values of the measurement and calculation correlated by 7%, except for the HIMRT plan at a depth of 1 mm. The uncertainty in the calculation accuracy of the surface dose of RTPS is well-known.^24^ It has been reported that the CCC and AAA algorithms cannot correctly calculate the absorbed dose at a depth of 1 mm.^24^ However, the calculation accuracy within the 3–5 mm depths was relatively better, and it was reported that the hair follicle exists at a depth of 3.5–4.2 mm from the skin surface.^25,26^ Based on the measurement results and the literatures,^25,26^ we defined the region between 3 and 5 mm from the skin surface as the scalp region and evaluated the dose parameters in this study.

A previous study investigated the dosimetric comparison of 3DCRT plans with 6 and 15 MV during WBRT and demonstrated that 15 MV beams achieved a considerably reduced scalp dose.^19^ The present study revealed that high-energy 10-MV beams were useful for markedly reducing scalp doses during 3DCRT and VMAT. The HIMRT plans were created with only 6 MV beams because tomotherapy is a single-energy X-ray machine. However, the ability of HIMRT to decrease the scalp dose was superior to that of VMAT with the same energy 6 MV in terms of mean scalp dose although 10MV-VMAT achieved the highest dose reduction. A limitation for HIMRT is the irradiation time. The average time over 30 patients exceeded 10 min, owing to the use of a 10 mm jaw size to improve the scalp-sparing. Oppositely, the VMAT plan took approximately 2 min for the irradiation.

We investigated the dose–volume parameters (V5Gy, V10Gy, V15Gy, V20Gy, V24Gy, and V30Gy) for the scalp. No significant differences were observed among the five plans in the scalp volume receiving 20 Gy or less, but HIMRT and VMAT substantially reduced the scalp volume receiving higher doses, such as 24 or 30 Gy. The trend of the dose reduction was similar to the mean scalp dose, but V30Gy of HIMRT was comparable to that of 10MV-VMAT. According to a previous study,^11^ WBRT using 11-field IMRT completely prevented alopecia in BM patients, and the average dose to the scalp was 16.3 Gy. In the aforementioned study, V24Gy and V30Gy of the scalp were evaluated as factors associated with alopecia, and the average volumes were 9.8 and 0.1 cc, respectively. A direct dosimetric comparison between the present and aforementioned results was difficult because the PTV margin in the previous study was 0 mm, and the scalp was defined as the region at a 5 mm depth from the skin surface. In clinical practice, a PTV margin of 5 or 10 mm is commonly used. However, a previous study reported that the PTV margin of 1 mm was sufficient for the treatment of the head and neck site if image-guided radiotherapy and an immobilization mask were available for the patient positioning.^27^ The results in this study showed that the mean scalp doses of HIMRT and 10MV-VMAT were 19.7 and 18.5 Gy, and the volumes of V24Gy and V30Gy were 7.1 and 4.2 cc and 0 and 0 cc, respectively, when the PTV margin was 1 mm. The results were comparable to that in the previously discussed study.^10^ We investigated the dose for scalp region that was dominantly decreased by the IMRT optimization. 10MV-VMAT achieved the highest dose reduction at the front, lateral, and back regions. However, the doses at top region in HIMRT were remarkedly lower than that in 10MV-VMAT. This may be because of the helical fashion, in which tomotherapy changes in intensity of the beam slice-by-slice according to craniocaudal coordinates.

Notably, this study did not evaluate the actual clinical outcome for the patients. We should have revealed how much the dose reduction of the scalp contributed to preventing alopecia and improving the QOL of the BM patients. Therefore, we are currently conducting a multi-institutional cohort study to explore the clinical benefit of HIMRT and VMAT during WBRT based on this study. The planning study’s limitation is that it did not attempt to spare the hippocampus. According to few studies, IMRT methods can achieve simultaneously scalp- and hippocampus-sparing.^13,14^ For the future cohort study, it would be crucial to incorporate hippocampus-sparing into scalp-sparing WBRT plans for minimizing scalp and neurocognitive side-effects.

## Conclusion

This study demonstrated the dosimetric efficacy of scalp-sparing in WBRT by comparing HIMRT with two energies of VMAT using the same patient cohort. HIMRT demonstrated the superior scalp-sparing than 6MV-VMAT. However, 10MV-VMAT achieved considerably lower scalp doses.
